# Helmet regulation in Vietnam: impact on health, equity and medical impoverishment

**DOI:** 10.1136/injuryprev-2015-041650

**Published:** 2016-01-04

**Authors:** Zachary Olson, John A Staples, Charles Mock, Nam Phuong Nguyen, Abdulgafoor M Bachani, Rachel Nugent, Stéphane Verguet

**Affiliations:** 1 School of Public Health, University of California, Berkeley, California, USA; 2 Department of Medicine, University of British Columbia, Vancouver, British Columbia, Canada; 3 Harborview Injury Prevention and Research Center, Seattle, Washington, USA; 4 Department of Global Health, University of Washington, Seattle, Washington, USA; 5 Department of Surgery, University of Washington, Seattle, Washington, USA; 6 World Health Organization, Hanoi, Viet Nam; 7 Johns Hopkins International Injury Research Unit, Department of International Health, Johns Hopkins Bloomberg, School of Public Health, Baltimore, Maryland, USA; 8 Department of Global Health and Population, Harvard T.H. Chan School of Public Health, Boston, Massachusetts, USA

## Abstract

**Background:**

Vietnam's 2007 comprehensive motorcycle helmet policy increased helmet use from about 30% of riders to about 93%. We aimed to simulate the effect that this legislation might have on: (a) road traffic deaths and non-fatal injuries, (b) individuals’ direct acute care injury treatment costs, (c) individuals’ income losses from missed work and (d) individuals’ protection against medical impoverishment.

**Methods and findings:**

We used published secondary data from the literature to perform a retrospective extended cost-effectiveness analysis simulation study of the policy. Our model indicates that in the year following its introduction a helmet policy employing standard helmets likely prevented approximately 2200 deaths and 29 000 head injuries, saved individuals US$18 million in acute care costs and averted US$31 million in income losses. From a societal perspective, such a comprehensive helmet policy would have saved $11 000 per averted death or $830 per averted non-fatal injury. In terms of financial risk protection, traffic injury is so expensive to treat that any injury averted would necessarily entail a case of catastrophic health expenditure averted.

**Conclusions:**

The high costs associated with traffic injury suggest that helmet legislation can decrease the burden of out-of-pocket payments and reduced injuries decrease the need for access to and coverage for treatment, allowing the government and individuals to spend resources elsewhere. These findings suggest that comprehensive motorcycle helmet policies should be adopted by low-income and middle-income countries where motorcycles are pervasive yet helmet use is less common.

## Introduction

Road traffic injury (RTI) accounts for a substantial and increasing burden of mortality, morbidity and healthcare costs in long-income and middle-income nations. Globally, road traffic is responsible for 1.3 million fatalities and 78 million non-fatal injuries per year.^1^ ^2^ In the Western Pacific, it is the leading cause of mortality for people aged 15–49.^3^ Direct economic costs are estimated to exceed $500 billion worldwide and are anticipated to grow in tandem with motorisation of the developing world.^2^ ^4^ Importantly, the potentially substantial medical out-of-pocket (OOP) costs associated with traffic injury may result in catastrophic expenditures (expenditures that crowd out a significant portion of household expenditures) and subsequent impoverishment.^5^

In response to the growing burden of traffic injury, the government of Vietnam passed a comprehensive motorcycle helmet use legislation in 2007. This legislation expanded mandatory helmet use to all riders on all roads, substantially increased penalties for helmet non-use and made provisions for increased enforcement.^6^ As a result, helmet use increased from 30% of riders to 93% within months.^7^ ^8^ Studies in other settings have examined the influence of helmet use policies on aggregate population health, but the distribution of benefits and equity improvements resulting from such changes in regulation remains understudied and uncertain.^9^ ^10^

Traffic injury can lead to substantial and potentially impoverishing health expenditures.^5^ Legislation mandating helmet use is one non-health sector policy that may protect individuals against this financial risk. In nations with universal health coverage, helmet regulation may have the additional advantage of reducing government traffic injury treatment expenditures and thus liberate spending for other health conditions. Defining the magnitude of the health and financial benefits attributable to Vietnam's comprehensive helmet policy might bolster the case for a similar policy in neighbouring countries such as Cambodia and in other low-income and middle-income countries.

Extended cost-effectiveness analysis (ECEA) incorporates the dimensions of equity and financial risk protection (FRP) into economic evaluation.^11–13^ In this paper, we used a simulation model to perform an ECEA that examines the influence that Vietnam's 2007 helmet legislation is anticipated to have had on: (a) road traffic deaths and non-fatal injuries, (b) individuals’ direct acute care injury treatment costs, (c) individuals’ income losses from missed work and (d) FRP for those individuals.

## Methods

### Design

For the era of interest, the annual number of non-fatal traffic injuries reported by Vietnam's National Traffic Safety Committee is not disaggregated by road user category and generally lacks consistency and credibility (eg, the 10 300 non-fatal road traffic injuries reported by police in 2007 are dramatically different from the 445 000 non-fatal road traffic injuries noted in health data reports from the same year).^14^ Recognising this, we chose to develop a model that uses secondary data to simulate the benefits that might be expected following the 2007 comprehensive helmet policy. After ensuring our model was consistent with previously reported reductions in total road traffic deaths,^6^ ^15^ we performed an ECEA to estimate the distribution of health benefits and costs across income groups. Conceptually, our study period includes a 1-year prepolicy baseline era (July 2006–June 2007), a 6-month transition period during which the majority of the helmet policy legislation was introduced and came into effect (June 2007–December 2007) and a 1-year postpolicy evaluation era (January 2008–December 2008).

### Setting

At the midpoint of our study, Vietnam was a lower-middle income country with a population of about 84 million and a per capita gross domestic product of about US$1200.^16^ About 95% of registered vehicles were motorised two-wheeled vehicles.^17^ The incidence of road traffic deaths prior to the 2007 helmet use legislation was estimated to be about 14 per 100 000 people per year.^14^ About 55% of healthcare costs were paid out of pocket.^18^ ^19^

Prior to 2007, Vietnam had limited motorcycle helmet legislation with incomplete implementation and enforcement. A comprehensive motorcycle helmet legislation that made helmet use compulsory for all motorcycle riders and passengers on all roads was introduced in June 2007, came into force for government workers in September 2007 and came in force for the general public in December 2007.^6^ Legislation introduced in September 2007 increased fines for helmet non-use from US$2–5 to US$11–22 per offence, the latter representing about 30% of the average monthly income per capita.^6^ ^20^ At that time, the majority of Vietnamese households were willing to pay the average market price of US$17 for a standard helmet.^21^

### Variables

In our simulation study, all input parameters were abstracted from academic studies and from reports issued by governmental and non-governmental agencies (table 1; online supplementary table S1). Output estimates of primary interest include traffic deaths averted, non-fatal traffic injuries averted, individuals’ OOP acute care medical costs averted and individuals’ income losses averted during the 1-year postpolicy era. Costs were viewed from the individual perspective including both OOP acute care costs and income losses. Estimation of subacute and chronic outpatient medical costs was not possible as reliable input parameters were not available. All costs were expressed in 2012 US dollars and were converted using consumer price indices and exchange rates as reported by the World Bank World Development Indicators.^16^

**Table 1:** Model input parameters*

Parameter	Estimate (range)	References
Population of Vietnam	84 000 000	16
Prepolicy RTI deaths	13 000	14
Prepolicy non-fatal RTIs	445 000	14
Proportion of RTI deaths attributable to motorcycles (%)	58 (51–73)	9 21 22
Proportion of non-fatal RTIs attributable to motorcycles (%)	59 (51–75)	7–9 21 22
Proportion of non-fatal motorcycle RTIs with head injury (%)	21 (10–32)	23
Prepolicy helmet use (%)	30 (20–40)	7 8
Postpolicy helmet use (%)	93 (83–98)	8
Average direct acute-care cost of non-fatal RTI with a helmet (US$)	436 (366–506)	23
Average direct acute-care cost of non-fatal RTI without a helmet (US$)	559 (416–702)	23
Expected increase in treatment cost for 10$ increase in income (%)	1	23
Income loss (weeks)	32	22
Mean per capita income, by quintile (US$)	305, 530, 777, 1185, 2730	24
Motorcycle ownership distribution by quintile (%)	20, 35, 54, 73, 94	25
RR of death, helmet vs no helmet	0.58 (0.50–0.79)	26
RR of injury, helmet vs no helmet	0.31 (0.25–0.66)	26
Per capita cost of policy implementation (US$)	0.29	Ref. 27 (correspondence from Dan Chisholm)

*Online supplementary appendix table S1 provides the detailed rationale and additional sources for selection of point estimates and ranges.

RTI, road traffic injury.

### Major assumptions

According to the National Vietnam Traffic Safety Committee, the number of registered motorcycles increased from 21 million in 2007 to 25 million in 2008; yet, for simplicity, our model makes the assumption that the number of registered motorcycles remained static at the prepolicy level during the study period.^15^ This assumption makes our estimates more conservative but substantially improves interpretability and generalisability. In our main analysis we assumed that the effectiveness of motorcycle helmets in Vietnam was equivalent to published estimates from high-income countries. Major concerns have been raised regarding the proliferation of substandard helmets in Vietnam.^28–30^ Local data regarding the effectiveness of substandard helmets was not available, and so we chose to address this crucial issue in a separate sensitivity analysis. For our main analysis, we assumed that the distribution of traffic deaths and non-fatal injuries across income quintiles reflected the distribution of motorcycle ownership across quintiles as obtained from the Vietnamese Demographic and Health Survey (DHS), an assumption that was further explored in sensitivity analyses.^25^

### Consequences for health

To simulate the impact on health consequences, we first estimated the number of traffic deaths and non-fatal head injuries that were attributable to motorcycles in the 1-year baseline era as well as the prepolicy proportion of motorcycle riders using helmets. Helmet effectiveness (expressed as the RR of head injury among helmeted riders compared with the risk among non-helmeted riders) was estimated using published ORs. By accounting for the increase in the proportion of helmeted riders following the comprehensive helmet policy, we simulated the number of deaths and head injuries averted within each quintile during the 1-year postpolicy evaluation era (see online supplementary appendix tables S3-S4 and equations S1-S4).

### Consequences for cost and affordability

We simulated the OOP acute care costs averted by the policy by subtracting the expected OOP costs of hospitalisation in the postpolicy era from the expected OOP cost in the baseline era. The expected cost was derived from published estimates on average cost of injury with and without a helmet, which takes into account variation in severity and type of injury based on helmet usage.^23^ These changes in costs were then multiplied by the estimated change in incidence of motorcycle injuries (see online supplementary appendix equation S5).

Empirical research has shown variation in average direct acute care cost of treatment by income group in Vietnam.^23^ We derived the average direct acute care cost of treatment in each income quintile by combining the estimated quintile-specific monthly income per capita with the reported 1% increase in traumatic brain injury (TBI) treatment cost for every $10 increase in monthly income per capita.^23^ ^24^ We calculated income losses by multiplying monthly per capita income by the Vietnamese average of 8 months away from work following TBI.^22^

We calculated two measures of FRP: cases of poverty averted and catastrophic health expenditures averted. Both measures of FRP reflect the reduction in financial hardship that may occur when an injury is averted or when the injury treatment cost is reduced. Cases of poverty averted were defined as the number of individuals who would no longer fall below the national poverty line due to traffic injury as a result of the helmet policy. In our base case model, the poverty line is such that 21% of the population lives in poverty.^16^ Cases of catastrophic health expenditures averted were defined as the number of people who would no longer be paying more than 25% of their per capita annual income on direct acute care costs as a result of the policy. The threshold for a catastrophic health expenditure varies depending on the literature but generally lies between 2.5% and 15% of household income or 10% and 45% of disposable income.^31^ Using a population of P individuals with a certain income distribution,^i^ we multiply the number of injured before an intervention in each quintile by the probability that they will face poverty or catastrophic health expenditure. We then do the same estimate based on the postintervention injury rate and costs. By subtraction we see how many cases of poverty or catastrophic health expenditure were averted in the population (see online supplementary appendix equation S6).

We approximated the governmental cost of implementing the comprehensive helmet legislation in Vietnam by multiplying the estimated per capita comprehensive helmet legislation implementation costs in Southeast Asia (including legislation and programme management, media, enforcement and helmet purchase) by the population of Vietnam.^27^ ^ii^

### Sensitivity analysis

We performed a univariate sensitivity analysis on key model inputs to test their influence on our findings. Upper and lower bounds for the inputs were obtained from published studies wherever possible and were otherwise derived based on available data or plausibly estimated (see online supplementary appendix and table S1). One critical sensitivity analysis explored the impact of substandard helmets in Vietnam, accounting for less safe designs (half-head or cap style), failure to meet quality standards and inadequate fastening of chin straps (Supplementary table S6). Each safety deficit was assumed to half the RR reduction for death or injury provided by the helmet, and this was combined with the approximate population prevalence of each deficit to estimate a lower bound of population-level helmet effectiveness (see online supplementary appendix, table S1 and figures S9).

We also performed an additional set of sensitivity analyses to evaluate the influence of model input distributional assumptions on the distribution of health and financial benefits across income quintiles (see online supplementary appendix and table S2). The distribution of motorcycle deaths and non-fatal injuries across quintiles, the distribution of prepolicy helmet use and the distribution of postpolicy helmet use across quintiles were varied in these analyses first alone and then by multivariate sensitivity analysis.

## Results

Assuming helmet effectiveness is equivalent to that in high-income countries, our simulation estimates that the 2007 comprehensive helmet policy might have prevented approximately 2200 deaths and 29 000 head injuries, saved individuals US$18 million in direct acute care costs and averted US$29 million in individual income losses in the year following its introduction Table 2. We estimate that countrywide implementation of the helmet policy cost the government US$24 million, although this was offset by an unknown amount of government revenue arising from increases in fines and enforcement.^27^ From a government perspective that accounts for implementation costs only, the helmet policy is estimated to cost about US$11 000 per death averted or US$800 per non-fatal injury averted. From a societal perspective (which sums individuals’ OOP direct acute care cost savings, individuals’ averted income losses and the government's implementation costs), the comprehensive helmet policy saved approximately US$11 000 per death averted or US$800 per non-fatal injury averted.

**Table 2:** Estimated reduction in death, injury and cost

	Prepolicy estimate (attributable to motorcycles)	Estimated absolute reduction (range*)	Estimated relative reduction, % (range*)
Deaths	7400	2200 (1000–2700)	29 (14–37)
Non-fatal head injuries	54 100	29 000 (12 700–44 500)	54 (23–82)
Direct acute care costs for non-fatal head injuries (million US$)	35	18 (8–28)	52 (24–81)
Income losses following death or non-fatal head injury (million US$)	63	29 (11–40)	46 (18–64)
Direct acute care costs plus income losses (million US$)	98	48 (24–72)	49 (24–73)

*Values in parentheses represent lower and upper bounds obtained on univariate sensitivity analyses.

**Figure 1:**
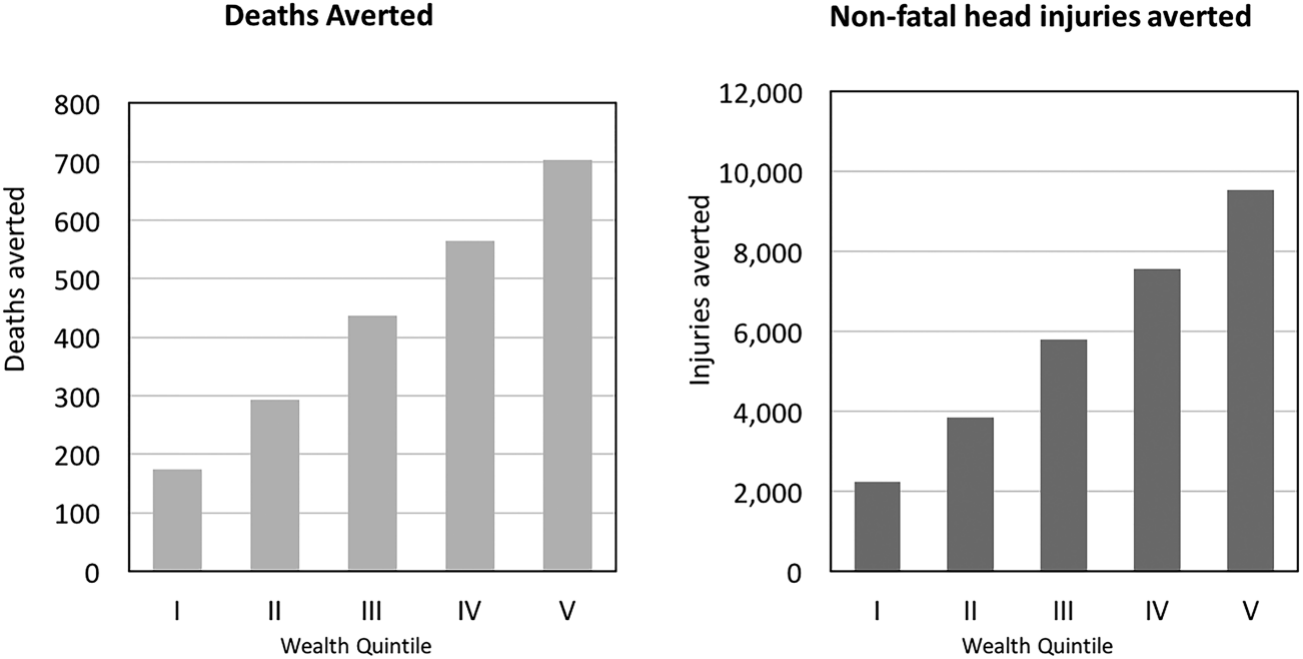
Deaths averted by income quintile (I, poorest, V, richest).

**Figure 2:**
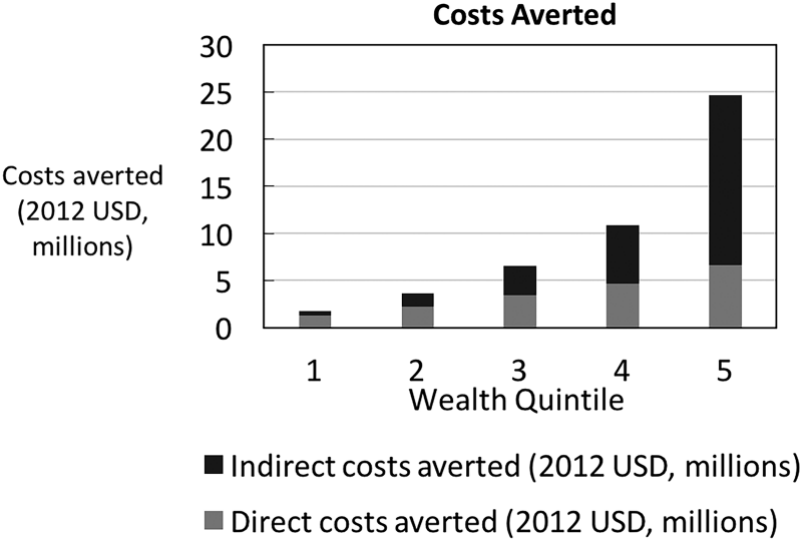
Out-of-pocket costs averted by income quintile (1, poorest, 5, richest).

**Figure 3:**
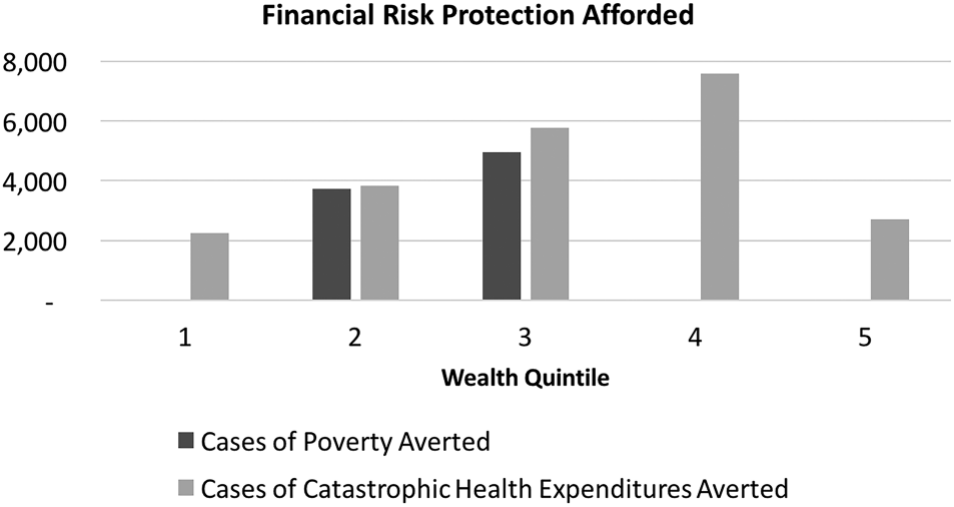
Financial risk protection afforded by income quintile (1, poorest, 5, richest).

The main distributional analysis assumes that the distribution of traffic injury reflects the distribution of motorcycle ownership across income quintiles and finds that the wealthiest quintiles own the greatest number of motorcycles and thus accrue a larger share of the health and financial benefits (in absolute terms) from the 2007 helmet policy (figure 1). In terms of FRP, traffic injury is so expensive to treat that any injury averted would necessarily entail a case of catastrophic health expenditure averted (figures 2 and 3; That is to say, both before and after the policy, traffic injury leads to health expenditures that exceed 25% of per capita income amounting to over 22 000 cases of catastrophic health expenditure averted. The helmet legislation is likely to help avert poverty for those in the second and third income quintiles amounting to nearly 11 000 cases of poverty averted. This is due to the fact that all those in the first quintile are already poor, and the cost is not so high that those in the fourth and fifth quintiles will be thrust into poverty.

### Sensitivity analysis

Lower and upper values obtained on univariate sensitivity analyses are presented in table 3. The sensitivity analysis that accounted for substandard and inadequately fastened helmets yielded the lowest estimates of averted deaths and injuries, a finding that has clear implications for policy and enforcement. Deaths, injuries and OOP costs averted were extremely sensitive to variation in the proportion of motorcycle injuries anticipated to cause head injury. Direct acute care costs averted were also highly sensitive to variation in the average acute care costs for helmeted and non-helmeted crash victims. These univariate sensitivity analyses, along with those for cases of poverty averted and catastrophic health expenditures averted, are presented graphically in the online supplementary figures S1–S5 and S9.

Distributional sensitivity analyses demonstrate that the distribution of health benefits is highly sensitive to variation in the distribution prepolicy of motorcycle injury across quintiles. Both health and financial benefits accrue disproportionately to the poor under conditions of perfectly equitable prepolicy motorcycle injury and death, a finding that is amplified when occurring in conjunction with highly inequitable prepolicy helmet use (with highest use among the wealthy) and perfectly equitable postpolicy helmet use (see online supplementary appendix and figures S4–S8).

## Discussion

Assuming helmet effectiveness equivalent to that in high-income countries, our simulation estimates that the 2007 comprehensive helmet policy prevented approximately 2200 deaths and 29 000 head injuries, saved individuals US$18 million in direct acute care costs and averted US$29 million in individual income losses in the year following its introduction. The combination of anticipated health and financial benefits make a comprehensive helmet policy strongly dominant to the prepolicy status quo. These findings suggest that similar comprehensive legislation and enforcement should be enacted in countries where motorcycles are pervasive yet helmet use is less common.

Importantly, the simulated relative reduction in motorcycle crash deaths fell from 29% to 14% after accounting for the proliferation of less effective helmets in Vietnam. Policymakers wishing to enact an effective comprehensive helmet law might wish to make provisions for adequate regulatory enforcement among manufacturers, retailers and motorcycle riders to ensure helmets are of adequate quality and appropriately fastened in order to maximise the health and financial benefits of their efforts.

The results of our ECEA suggest that the wealthy likely accrued a large share of the absolute health and financial benefits resulting from helmet use legislation. This finding was dependent on our assumption that the risk of RTI tracked with motorcycle ownership. In contrast, under all conditions tested we found that the legislation likely prevented a greater number of motorcycle-related cases of poverty among the near poor and middle-income quintiles. This supports the conclusion that injury prevention is also poverty prevention among individuals of lesser wealth. In settings with universal health insurance, cost savings from a comprehensive helmet policy (potentially substantial, as the wealthy are known to use a disproportionate share of public health care) might also be liberated for use on other health policy priorities.^31^

The validity of our model's estimates is supported by the results of prior research. Our analysis anticipates a 29% reduction in motorcycle traffic deaths and a 17% reduction in total traffic deaths, results that are similar to the 36% reduction in motorcycle traffic deaths generally anticipated with helmet legislation and the 18% reduction in total traffic deaths reported in Vietnam in the year following introduction of the helmet legislation.^6^ ^10^ Our results are also in harmony with the results of other regional evaluations of helmet use legislation.^33–35^

Our analysis presents several limitations that relate to our model and its inputs. First, we emphasise that our modelling study estimated the anticipated effectiveness and cost-effectiveness of the 2007 comprehensive helmet policy in Vietnam but did not measure the benefits or costs directly. To our knowledge, the observed benefits and costs of this policy have not been clearly articulated in the published academic literature despite the crucial importance of these values to an evaluation of policy success. Second, many of our inputs (including prepolicy deaths and injuries attributable to motorcycles, acute care costs and policy implementation costs) were not directly available and had to be derived or estimated from published reports. The predominant use of academic and non-governmental reports in preference to government surveillance data prioritises data quality but might diminish the local applicability of our results. Third, our main analysis ignored the influence of substandard helmets in Vietnam. For this reason, we pursued a sensitivity analysis examining this issue limited by the absence of reliable estimates of the relative effectiveness of the substandard helmets, particularly in a setting with relatively low traffic speeds.^36^ ^37^

Our analysis is also limited by a number of assumptions we made in constructing our model. We assumed a constant number of motorcycles on the road before and after the policy, rendering our estimated benefits more conservative, interpretable and generalisable.^38^ We ignored changes in the prevalence of speeding and alcohol use, increased enforcement of non-helmet laws, changes in road maintenance and congestion and other secular trends. For our cost estimates, we do not account for a potential increase in non-head injuries among riders whose lives were saved by helmet use as the simulated number of deaths averted represents <10% of the simulated number of injuries averted, and this influence is anticipated to be minimal. Lastly, we had insufficient information to estimate the increases in individuals’ costs and government revenue resulting from improvements in enforcement and increased fines resulting from the helmet policy. The potential for impoverishment due to helmet infraction fines was assumed to be uncommon and relatively inconsequential.

Our results suggest that Vietnam's 2007 helmet legislation was cost-effective. Our ECEA analysis suggests that large health and financial benefits accrued to the wealthy, yet the policy also provided significant health benefits and substantial FRP to Vietnam's poorest citizens. Policymakers wishing to account for such effects may want to use ECEA to understand the likely influence of policy on equity.

**What is already known on the subject**

- Helmet usage is a cost-effective way to prevent traumatic brain injury.
- Vietnam's helmet law successfully increased helmet usage among Vietnamese motorbike riders.

**What this study adds**

- An estimation of the anticipated distribution of the benefits of helmet usage across socio-economic groups.
- An estimation of the anticipated level of financial risk protection afforded under helmet regulation.
- An estimation of the anticipated impact of substandard helmets on the population level benefits of a comprehensive motorcycle helmet law.

**Green cross safety awards**

The US National Safety Council (NSC) presented awards to the Air Force Safety Center, to United Airlines Corporate Safety and to Gary Smith at Nationwide Children's Hospital. The Air Force award was for real-time evaluations that apparently resulted in a 29% fall in ‘mishaps’. After United and Continental Airlines merged, new regulations required them to implement a safety management system. Two years later injuries were reduced by 11%. Finally, Dr Smith is a researcher and advocate for paediatric injury prevention, especially with respect to consumer product safety. He played a key role in drafting the 2012 National Action Plan for Child Injury Prevention.

**Occupational safety modernises data collection**

The Occupational Safety and Health Administration (OSHA) issued a new rule in 2016 that applies ideas from behavioural economics to improve workplace safety.

The rule requires employers in high-hazard industries to send injury data to the agency's website. OSHA believes this will “encourage employers to increase their efforts to prevent” these injuries. The data will also enable employers to compare their safety performance against that of others. As a bonus, these data will be part of the largest publicly available data set on work injuries and thus benefit researchers.

**Pfizer drugs not to be used in executions**

Pfizer announced that none of its drugs can be used in lethal injections in executions. 20 other drug companies have this policy to avoid marketing difficulties. The policy has prompted several states to adopt furtive methods for getting these drugs. Other states have delayed executions while still others have chosen the electric chair, firing squads or the gas chamber as alternatives. *Comment*: One bizarre aspect of this issue hinges on whether drug substitutes might not meet quality standards causing ‘undue suffering’. That puzzler aside, the Pfizer decision comes against a backdrop of declining numbers of executions in the US; in 2015 there were 28 vs 98 in 1999.

## Supplementary Material

Web supplement

## Footnotes

i A proxy for individual income can be extracted from the income distribution of Vietnam derived from its gross domestic product per capita (US$1200 in 2012) and its Gini index (0.36).^16^ ^32^

ii This number was derived by Dan Chisholm, Jonathon Passmore, and Nguyen Phuong Nam using the same model cited.^2^,^7^
