# Supplementary material for: Helmet regulation in Vietnam: impact on health, equity and medical impoverishment
Source: Inj Prev. 2016 Jan 4;22(4):233–8. doi: 10.1136/injuryprev-2015-041650 (PMC4975813; doi:10.1136/injuryprev-2015-041650)
Supplement: Web supplement [file injuryprev-2015-041650-s1.pdf]

Supplementary Appendix

Helmet Regulation in Vietnam: Impact on health and medical impoverishment

by

Zachary Olson[1,*] , John A Staples[2,3,*], Charles Mock[3,4,5], Nam Phuong Nguyen[6], Abdulgafoor M Bachani[7], Rachel Nugent[4], Stéphane Verguet[8]

Author Affiliations

[1.] School of Public Health, University of California, Berkeley, USA

[2.] Department of Medicine, University of Washington, Seattle, USA

[3.] Harborview Injury Prevention and Research Center, Seattle, USA

[4.] Department of Global Health, University of Washington, Seattle, USA

[5.] Department of Surgery, University of Washington, Seattle, USA

[6]. World Health Organization, Viet Nam Country Office, 63 Tran Hung Dao Street, Hanoi, Viet Nam.

[7.] Johns Hopkins International Injury Research Unit, Department of International Health, Johns Hopkins Bloomberg, School of Public Health, Baltimore, USA

[8.] Department of Global Health and Population, Harvard T.H. Chan School of Public Health, Boston, USA

Correspondence:

Zachary Olson, School of Public Health, University of California, Berkeley. 3020 College Avenue, Unit 3. Berkeley, CA 94705. Telephone +1-202-713-9221 Email: zolson@berkeley.edu

[*] Both authors contributed equally to this paper.

Table S1. Parameters used for the extended cost-effectiveness analysis (ECEA) of helmet policy in Vietnam, with justification

Parameter	Estimate (Range)	Justification and References
Population of Vietnam	84,221,100	[12]. Data from 2007.
Pre-policy RTI deaths	12,800	[13]. Data from 2007.
Pre-policy non-fatal RTIs	445,048	[13]. Data from 2007.
Proportion of RTI deaths attributable to motorcycles	57.9% (51.3% - 72.7%)	Estimate from a post-policy verbal autopsy study of 1,061 RTI deaths performed in 2008-2009 [9]. Lower bound from a 2001 community-based survey describing proportion of non-fatal RTI attributable to motorcycles (cited in [19]). Upper bound from a 2004 analysis of that determined 72.7% of 7,915 vehicle collisions involved a motorcycle (cited in [18]).
Proportion of non-fatal RTIs attributable to motorcycles	59% (51.3% - 74.8%)	Estimate from a survey circa 2002 (cited in [7]); similar to estimate of proportion of RTI deaths attributable to motorcycles obtained from a post-policy verbal autopsy study of 1,061 RTI deaths performed in 2008-2009 [9]. Lower bound from a 2001 community-based survey describing proportion of non-fatal RTI attributable to motorcycles (cited in [19]). Upper bound from a 2010 prospective study that found 74.8% of 477 RTI hospital admissions were motorcycle riders [20]; similar to a 2004 analysis of that determined 72.7% of 7915 vehicle collisions involved a motorcycle (cited in [18]).

Proportion of non-fatal motorcycle RTIs with head injury	20.6% (9.5% - 31.7%)	Estimate derived from lower bound: If 9.5% of injured motorcyclists have a head injury during an epoch in which 93% of riders are wearing helmets and the relative risk of head injury comparing helmet users to non-helmet users is 0.31, an epoch in which 30% of riders are wearing helmets is likely to result in 20.6% of injured motorcyclists having head injuries (see Equations S1 and S2). Lower bound from a 2010 post-policy prospective study of 477 RTI admissions that found 34 of 357 motorcycle riders reported their head as the principally injured region [20]. Upper bound derived by adding to the estimate the difference between the estimate and lower bound.
Pre-policy helmet use	29.9% (20% - 40.1%)	Estimate from the weighted average of a 2005 population-based observational survey of 16,560 motorcyclists on 5 road categories [7]. Lower bound derived by subtracting from the estimate the difference between the upper bound and the estimate. Upper bound from non-weighted observational cross sectional data taken in November 2007 (just prior to full enforcement of the policy) from 110,677 motorcycle riders in 3 provinces [20].
Post-policy helmet use	92.5% (82.5% - 97.5%)	Estimate from non-weighted observational cross sectional data taken from 554,781 motorcycle riders in 3 provinces in 2008 - 2011 [8]. Lower bound derived by the arbitrary subtraction of 10% from the estimate. Upper bound derived by the arbitrary addition of 5% to the estimate.

Proportion of incorrectly fastened helmets	22%	Estimate taken from a survey of 377 motorcyclists at Taiwanese petrol-stations who reported a crash while wearing a helmet in the past year [37]. Note that only 1.5% of 554,781 motorcycle riders were observed with a completely unfastened helmet in a non-weighted observational cross sectional study of in 3 Vietnamese provinces in 2008 – 2011 [8], but that anecdotal evidence suggests unfastened or loosely fastened helmets are far more prevalent than that.
Proportion of less safe helmet designs	25%	Estimate derived from the prevalence of half-face, open-face, and cap style helmets acquired in a 2011 cross sectional roadside study in which 582 motorcyclists agreed to provide their helmet for standard quality testing in exchange for a new helmet [38]. Estimate of 56% of motorcyclists with half-coverage helmets taken from a survey of 377 motorcyclists at Taiwanese petrol-station with a crash while wearing a helmet in the past year [37]. Estimate of 88% of helmets worn improperly from a newspaper report that describes a survey of over 11,000 people [Vietnam News, 2008 INSERT REF NUMBER]
Proportion of helmets of substandard quality	81%	Estimate derived from the proportion of helmets failing at least one standard quality test among those acquired in a 2011 cross sectional roadside study in which 582 motorcyclists exchanged their current helmet for a new helmet [38].

Average direct acute-care cost of non-fatal RTI with a helmet (US$)	$436 ($366 – $506)	Estimate from a 2010 prospective study of 477 RTI admissions that stratified hospital mean costs by principal injured region and helmet use, converted to US$ [22]. Lower and upper bounds represent the 95% confidence intervals and were derived from the listed standard deviations [22].
Average direct acute-care cost of non-fatal RTI without a helmet (US$)	$559 ($416 - $702)	Estimate from a 2010 prospective study of 477 RTI admissions that stratified hospital mean costs by principal injured region and helmet use, converted to US$. Lower and upper bounds represent the 95% confidence intervals and were derived from the listed standard deviations [22].
Change in treatment cost for 10$ change in income	1%	[22]
Income lost	32 weeks	[21]
Per capita income distribution by quintile (US$)	$308, $558, $835, $1244, $2847	[23]
Motorcycle ownership by income quintile (%)	20%, 35%, 54%, 73%, 94%	[24]. Used to estimate the burden of motorcycle RTI injury and death within each quintile.

Relative risk of death, helmet versus no helmet	0.58 (0.50 – 0.79)	Point estimate and lower bound are derived using the odds ratio point estimate and lower 95% confidence interval from a 2008 meta-analysis [25]. Given the low absolute risk of death or injury among motorcycle riders, the odds ratio was assumed to be a reasonable estimate of the relative risk. Upper bound estimated by assuming a 50% relative reduction in population-level helmet effectiveness in the Vietnamese context (see Supplemental Figure S9).
Relative risk of injury, helmet versus no helmet	0.31 (0.25 – 0.66)	Point estimate and lower bound are derived using the odds ratio point estimate and 95% confidence interval from a 2008 meta-analysis [25]. Given the low absolute risk of death or injury among motorcycle riders, the odds ratio was assumed to be a reasonable estimate of the relative risk. Upper bound estimated by assuming a 50% relative reduction in population-level helmet effectiveness in the Vietnamese context (see Supplemental Figure S9).
Per capita cost of policy implementation (US$)	$0.29	[26]

Pre-policy registered motorcycles	21.2 million	[27]
Pre-policy registered motorcycles	25.2 million	.[27]
Pre-policy revenue from helmet infringements (US$, millions)	Unknown	No data. Fines US$2-5 per offence.

Table S2: Values used for the distributional sensitivity analysis[1]

	Income Quintile					Mean	Q5:Q1 Ratio
	I	II	III	IV	V		
**Distribution of motorcycle RTI deaths and non-fatal injuries (proportion borne by each income quintile)[2]**							
Input for main analysis (severely inequitable)[3]	7%	13%	20%	26%	34%	-	4.6
Moderately inequitable	15%	18%	20%	23%	25%	-	1.7
Perfectly equitable	20%	20%	20%	20%	20%	-	1
**Distribution of pre-policy helmet use**							
Input for main analysis (mildly inequitable)[4]	24%	27%	30%	33%	36%	30%	1.5
Moderately inequitable	10%	20%	30%	40%	50%	30%	5
Severely inequitable	6%	12%	24%	48%	60%	30%	10
**Distribution of post-policy helmet use**							
Input for main analysis (perfectly equitable)	93%	93%	93%	93%	93%	93%	1
Mildly inequitable	88%	90%	93%	95%	97%	93%	1.1
Moderately inequitable	84%	88%	93%	97%	100%	93%	1.2

1 Distributions estimated using plausible values except where specified as having been derived from a specific source.
2 Values listed here were used to create weights to distribute the total pre-policy motorcycle RTI deaths and injuries among quintiles.
3 For the main analysis, motorcycle RTI deaths and injuries were assumed to have a distribution among quintiles corresponding to probability of household motorcycle ownership [24].
4 Approximated using the relationship between helmet ownership and income [36].

Table S3, Equations S1 & S2: Calculating the proportion of non-fatal motorcycle RTIs with head injury

Table S3. Symbols and Definitions

Symbol	Definition
G_0, G_1	Number of motorcycle crashes resulting in non-fatal injury (pre-intervention, post-intervention)
E_0, E_1	Proportion of motorcycle crash injuries with head injury (pre-intervention, post-intervention)
H_0, H_1	Proportion of motorcycle riders wearing helmets (pre-intervention, post-intervention)
I_0, I_1	Number of head injuries (pre-intervention, post-intervention)
AR_H, AR_{NH}	Absolute risk of head injury (with helmet, without helmet)
RR	Relative risk of injury, helmet vs. no helmet ($RR = AR_H/AR_{NH}$)

(S1)

$$I0 = G_0 \times \mathrm{H0} \times \mathrm{AR}H + G_0 \times (1 - \mathrm{H0}) \times \mathrm{AR}_{NH}$$

$$I0 = G_0 \times \mathrm{H0} \times \mathrm{AR}H + G_0 \times (1 - \mathrm{H0}) \times \frac{\mathrm{AR}_H}{\mathrm{RR}}$$

$$I0 = G_0 \times \mathrm{AR}H \times [\mathrm{H0} + (1 - \mathrm{H0}) \times \frac{1}{\mathrm{RR}}]$$

Similarly $$I1 = G_1 \times \mathrm{AR}H \times [\mathrm{H1} + (1 - \mathrm{H1}) \times \frac{1}{\mathrm{RR}}]$$

(S2) Assuming helmet use reduces the risk of head injury in the event of a crash but has no influence on overall crash risk and no influence on the risk of additional non-fatal injuries to other body parts in the event of a crash (ie $G_0 = G_1$):

$$E_0 = \frac{I_0}{G}$$

$$E_0 = E_1 \times (\frac{I_0}{I_1})$$

$$E_0 = E_1 \times (\frac{[\mathrm{H}_0 + (1 - \mathrm{H}_0) \times \frac{1}{\mathrm{RR}}]}{[\mathrm{H}_1 + (1 - \mathrm{H}_1) \times \frac{1}{\mathrm{RR}}]})$$

Using equations S1 and S2, we are able to estimate the proportion of motorcycle crashes that result in head injury in the pre-policy period. Using the total RTI injuries, the

proportion of RTI injuries attributable to motorcycles, and the proportion of motorcycle crashes that result in head injuries allows estimation of I_0.

Table S4, Equations S3 and S4: Calculating the number of post-policy head injuries

Table S4. Symbols and Definitions

Symbol	Definition
P	Population at risk
H_0, H_1	Helmet use pre-intervention, post-intervention
I_0, I_1	Head injuries pre-intervention, post-intervention
AR_H, AR_{NH}	Absolute risk of injury with helmet, without helmet
RR	Relative risk of injury, helmet vs no helmet (RR = AR_H/AR_{NH})

(S3)

$$I_0 = \mathrm{P}\times\mathrm{H}_0\times\mathrm{AR}_H + \mathrm{P}\times(1-\mathrm{H}_0)\times\mathrm{AR}_{NH}$$

$$I_0 = \mathrm{P}\times[\mathrm{H}_0\times\mathrm{AR}_H + (1-\mathrm{H}_0)\times\mathrm{AR}_{NH}]$$

$$I_0 = \mathrm{P}\times[\mathrm{H}_0\times\mathrm{AR}_H + (1-\mathrm{H}_0)\times\frac{\mathrm{AR}_H}{\mathrm{RR}}]$$

$$\mathrm{AR}_H = I_0 \div \left(\mathrm{P}\times\left[\mathrm{H}_0 + \frac{(1-\mathrm{H}_0)}{\mathrm{RR}}\right]\right)$$

(S4)

$$I_1 = \mathrm{P}\times[\mathrm{H}_1\times\mathrm{AR}_H + (1-\mathrm{H}_1)\times\mathrm{AR}_{NH}]$$

$$I_1 = \mathrm{P}\times[\mathrm{H}_1\times\mathrm{AR}_H + (1-\mathrm{H}_1)\times\frac{\mathrm{AR}_H}{\mathrm{RR}}]$$

$$I_1 = \mathrm{P}\times\mathrm{AR}_H\times[\mathrm{H}_1 + \frac{(1-\mathrm{H}_1)}{\mathrm{RR}}]$$

$$I_1 = \mathrm{P}\times[\frac{I_0}{\left(\mathrm{P}\times\left[\mathrm{H}_0+\frac{(1-\mathrm{H}_0)}{\mathrm{RR}}\right]\right)}]\times[\mathrm{H}_1 + \frac{(1-\mathrm{H}_1)}{\mathrm{RR}}]$$

$$I_1 = [\frac{I_0}{\left(\left[\mathrm{H}_0+\frac{(1-\mathrm{H}_0)}{\mathrm{RR}}\right]\right)}]\times[\mathrm{H}_1 + \frac{(1-\mathrm{H}_1)}{\mathrm{RR}}]$$

Using equations S3 and S4, we are able to estimate the number of head injuries in the post-policy period.

Table S5, Equations S5 and S6: Calculating costs averted and financial risk protection.

Table S5. Symbols and Definitions

Symbol	Definition
I_0, I_1	Number of head injuries (pre-intervention, post-intervention)
H_0, H_1	Proportion of riders wearing helmets (pre-intervention, post-intervention)
D_0, D_1	Number of deaths (pre-intervention, post-intervention)
C_0, C_1	Average treatment cost (pre-intervention, post-intervention)
C_H, C_{NH}	Cost of injury (helmet, no helmet)
P	Population
n	Simulated number of individuals
y	Average Income
L	Poverty Line
FRP_P, FRP_C	Financial risk protection (Cases of poverty averted, catastrophic health expenditures averted)
I_0, I_1	Number of head injuries (pre-intervention, post-intervention)
H_0, H_1	Proportion of riders wearing helmets (pre-intervention, post-intervention)

(S5)

$$C_{0,1} = C_H \times H_{0,1} + C_{NH} \times (1 - H_{0,1})$$

$$OOP\ Direct\ Acute\ Care\ Costs\ Averted = C_0 \times I_0 - C_1 \times I_1$$

(S6)

$$FRP_P = \left(I_0 \times \frac{\sum_{i=1}^{P} \begin{cases} 1\ if\ y - C_0 < L \\ 0\ if\ y - C_0 > L \\ 0\ if\ y < L \end{cases}}{P} \right) - \left(I_1 \times \frac{\sum_{i=1}^{P} \begin{cases} 1\ if\ y - C_1 < L \\ 0\ if\ y - C_1 > L \\ 0\ if\ y < L \end{cases}}{P} \right)$$

$$FRP_C = \left(I_0 \times \frac{\sum_{i=1}^{P} \begin{cases} 1 \; if \; .25 \times y < C_0 \\ 0 \; if \; .25 \times y > C_0 \end{cases}}{P} \right) - \left(I_1 \times \frac{\sum_{i=1}^{P} \begin{cases} 1 \; if \; .25 \times y < C_1 \\ 0 \; if \; .25 \times y > C_1 \end{cases}}{P} \right)$$

Using equations S5 and S6 we are able to quantify the change in income and thus catastrophic expenditure from motorcycle accidents.

Table S6: Estimating the influence of poor quality and inadequately fastened helmets

	**Standard Helmets** (from Liu *et al* [25])	**Poor Quality and Inadequately Fastened Helmets** (estimates)
**Injury**		
**Relative Risk**	0.31	0.66
**Relative Risk Reduction**	0.69	0.35
**Death**		
**Relative Risk**	0.58	0.79
**Relative Risk Reduction**	0.42	0.21

Yu and colleagues performed a case-control study in Taiwan to estimate the influence of improper use on helmet efficacy [37]. Compared to non-helmeted motorcyclists, helmeted motorcyclists were less likely to have head injuries (odds ratio 0.22). Compared to motorcyclists wearing full-coverage helmets, those with half-coverage helmets were about twice as likely to have head injuries (odds ratio 2.57). Compared to motorcyclists with appropriately fastened helmets, those with loosely or unfastened helmets were also about twice as likely to have head injuries (odds ratio 1.94).

In Vietnam in 2007, we estimate that 80% of helmets were substandard, 25% had less safe designs (half-head, open-faced, or cap style), and 21.5% were inadequately applied or secured [37, 38]. These deficiencies are not mutually exclusive and may co-exist in any given helmet.

To estimate the influence of substandard and improperly applied helmets on the results of our simulation, we assumed that Vietnamese helmets, on average, provided half the relative risk reduction of helmets in high-income countries (see Table S2). The resulting values were used as in the univariate sensitivity analysis that varied the relative risk describing the efficacy of helmets.

Figure S1: Sensitivity of deaths averted to univariate model inputs

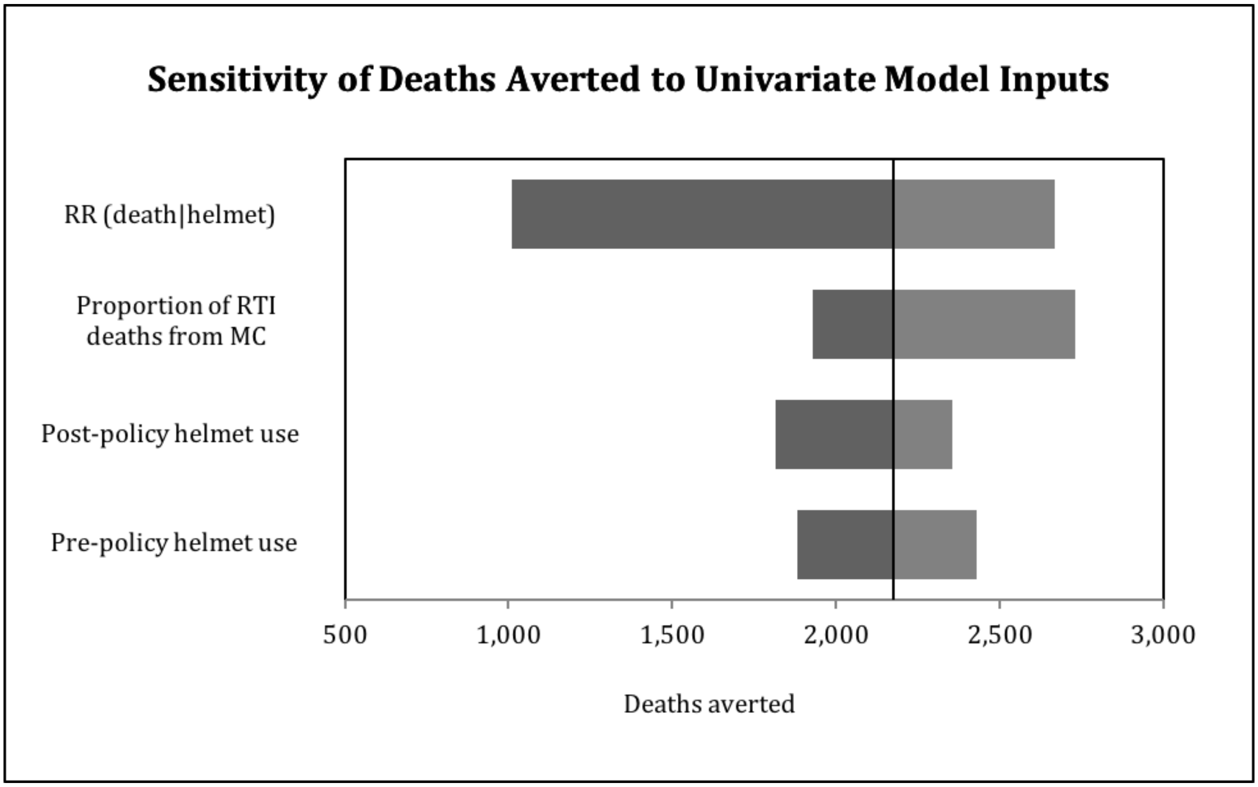

Figure S2: Sensitivity of injuries averted to univariate model inputs

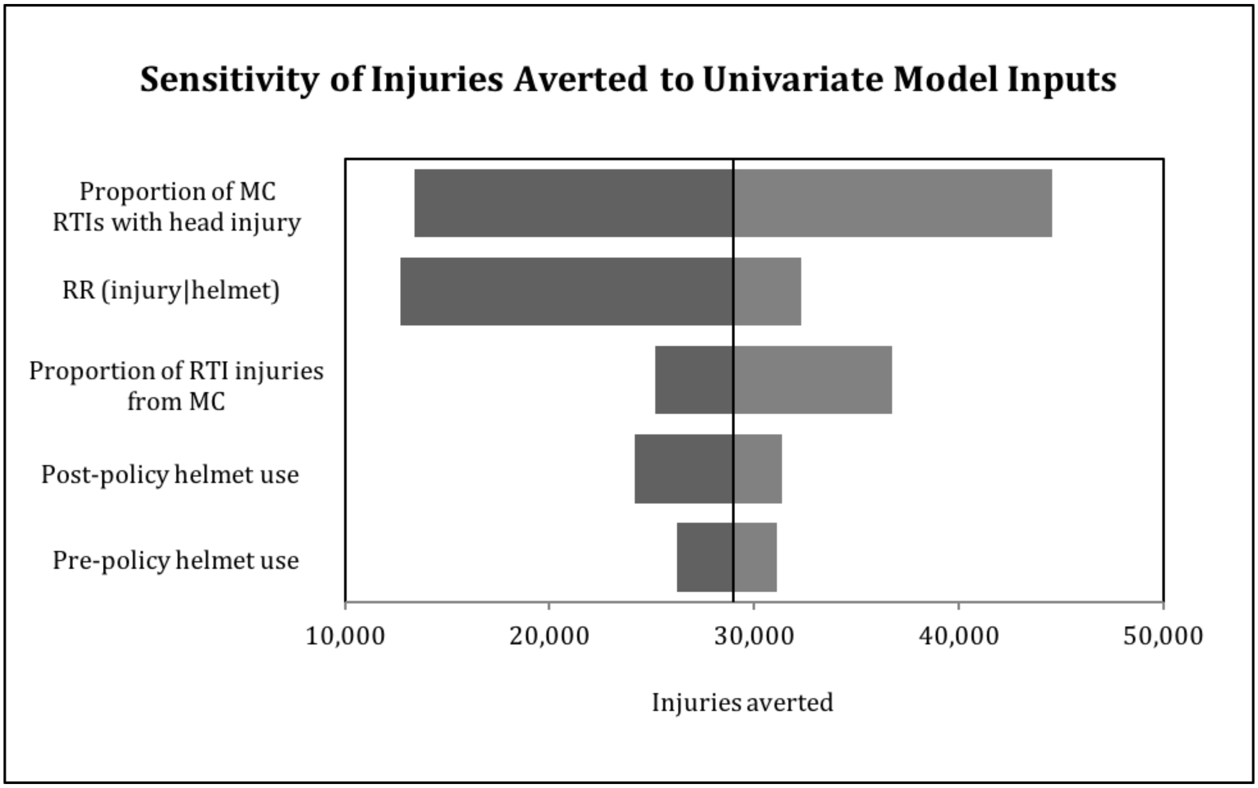

Figure S3: Sensitivity of direct acute-care costs averted to univariate model inputs

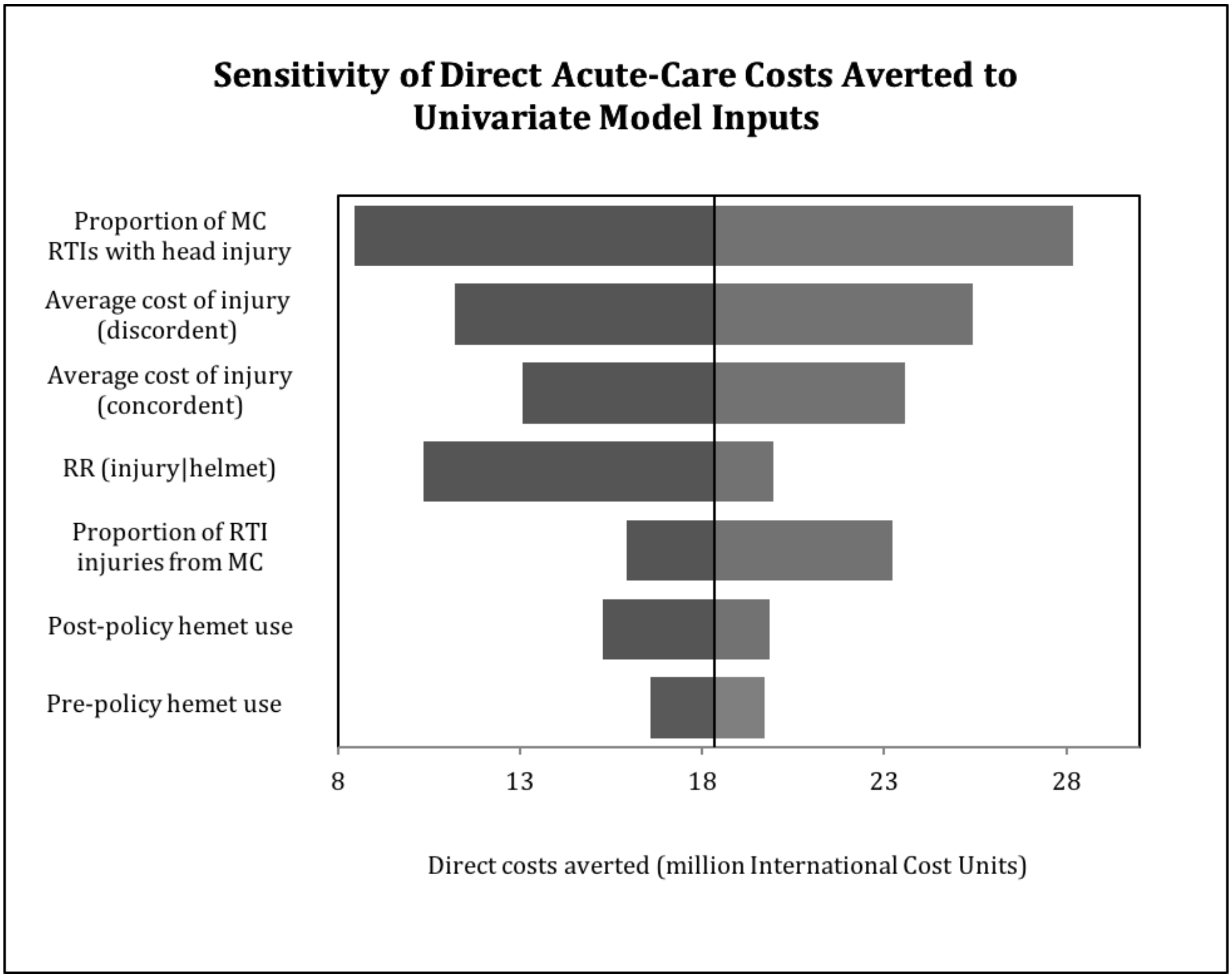

Figure S4: Sensitivity of cases of poverty averted to univariate model inputs

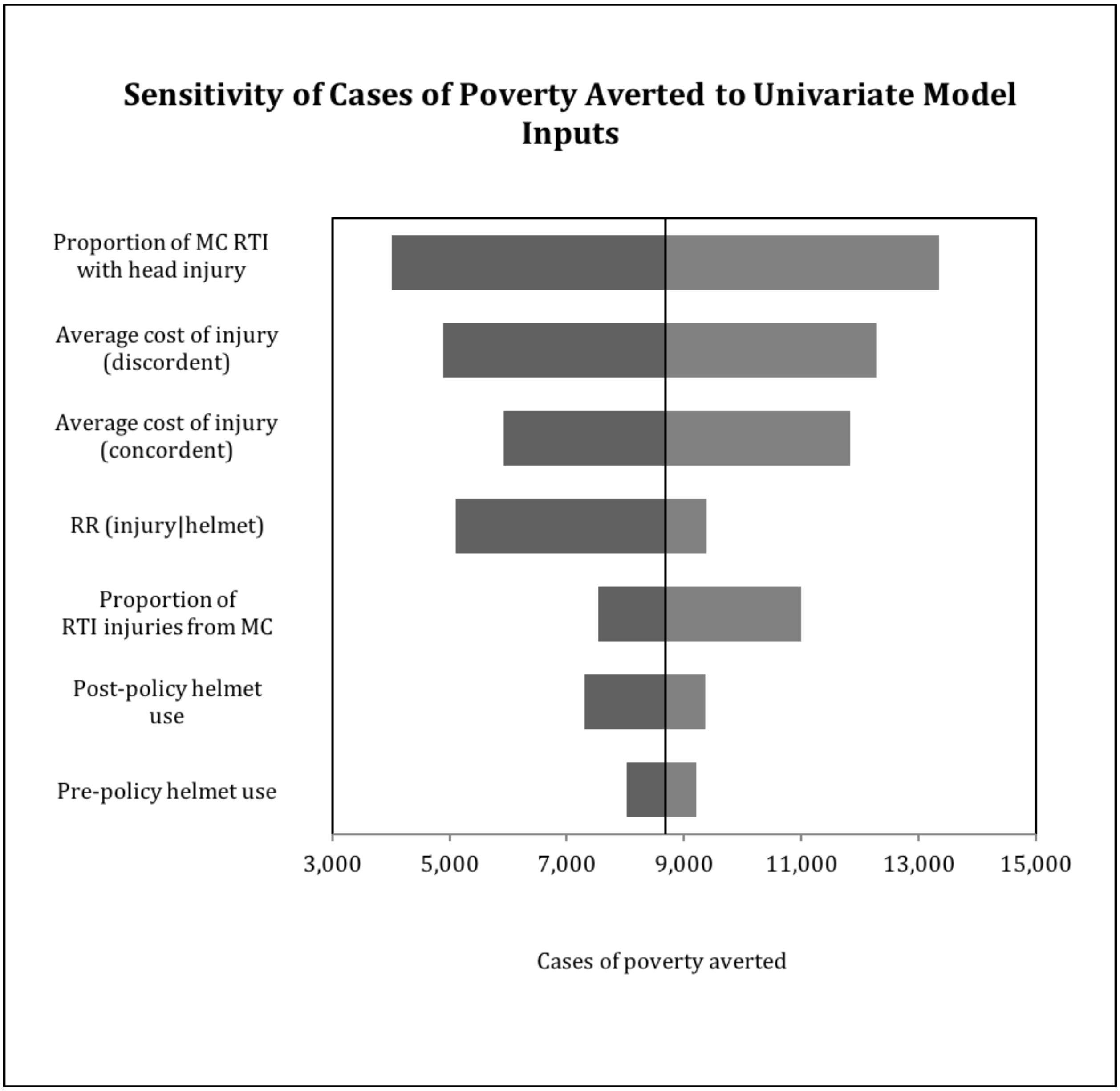

Figure S5: Sensitivity of catastrophic health expenditures averted to univariate model inputs

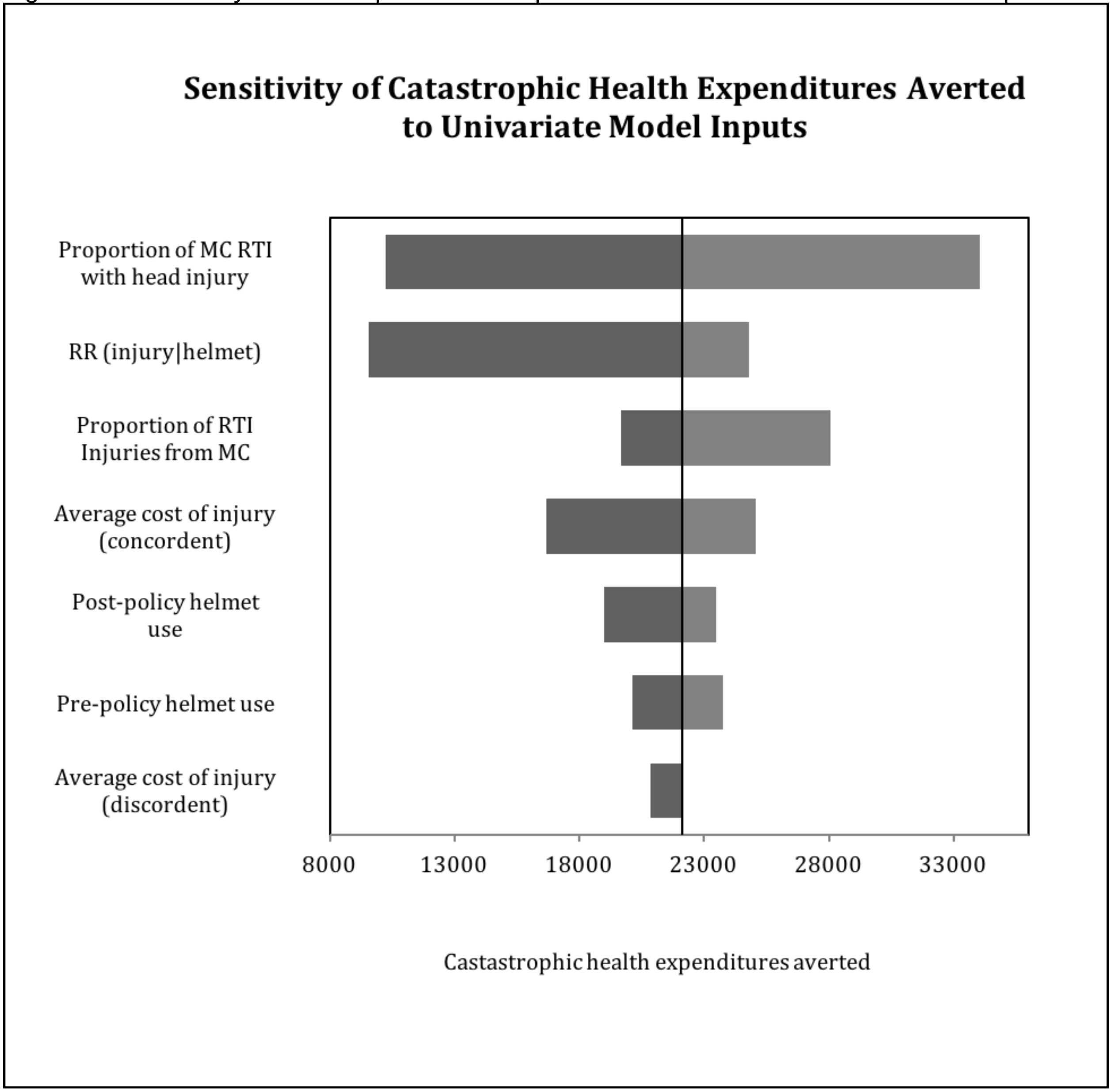

Figure S6: Sensitivity analysis assuming perfectly equitable motorcycle use across quintiles (I being poorest, V being richest). See table S2 for equitable assumptions.

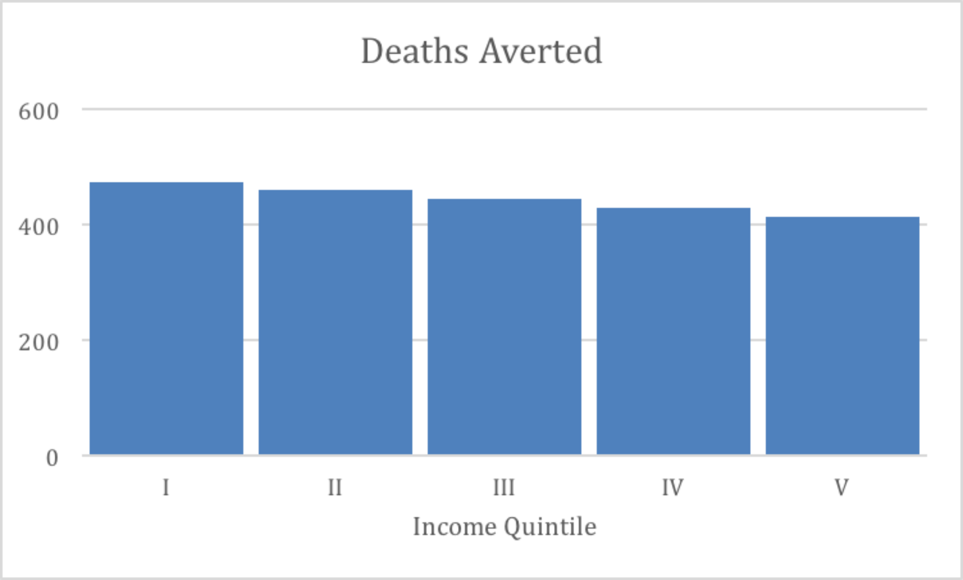

Figure S7: Sensitivity analysis assuming moderately inequitable motorcycle use, moderately inequitable pre-policy helmet use, and moderately inequitable post-policy helmet use across quintiles. (I being poorest, V being richest). See table S2 for moderately inequitable assumptions.

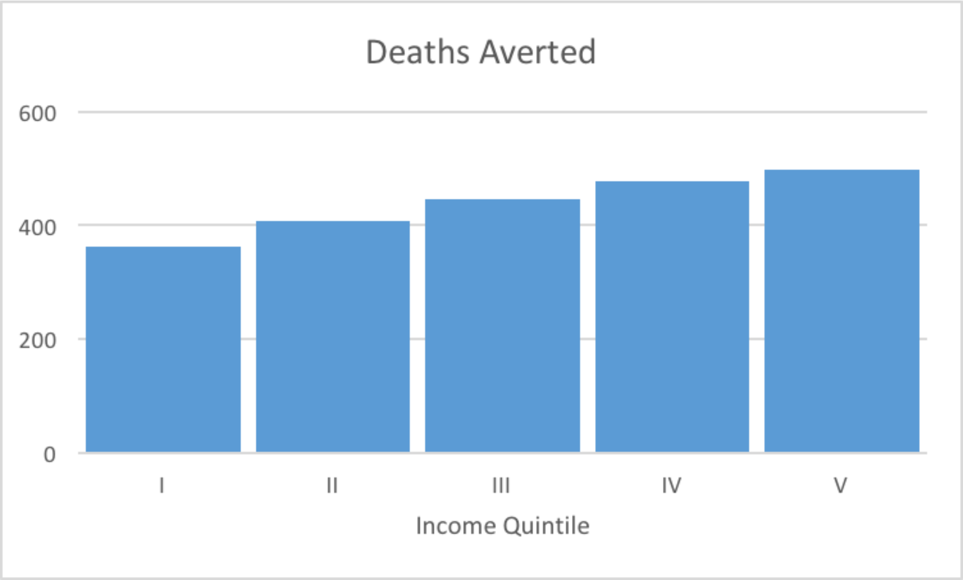

Figure S8: Sensitivity analysis assuming perfectly equitable motorcycle use, severely inequitable pre-policy helmet use, and perfectly equitable post-policy helmet use across quintiles. (I being poorest, V being richest). See table S2 for perfectly equitable assumptions.

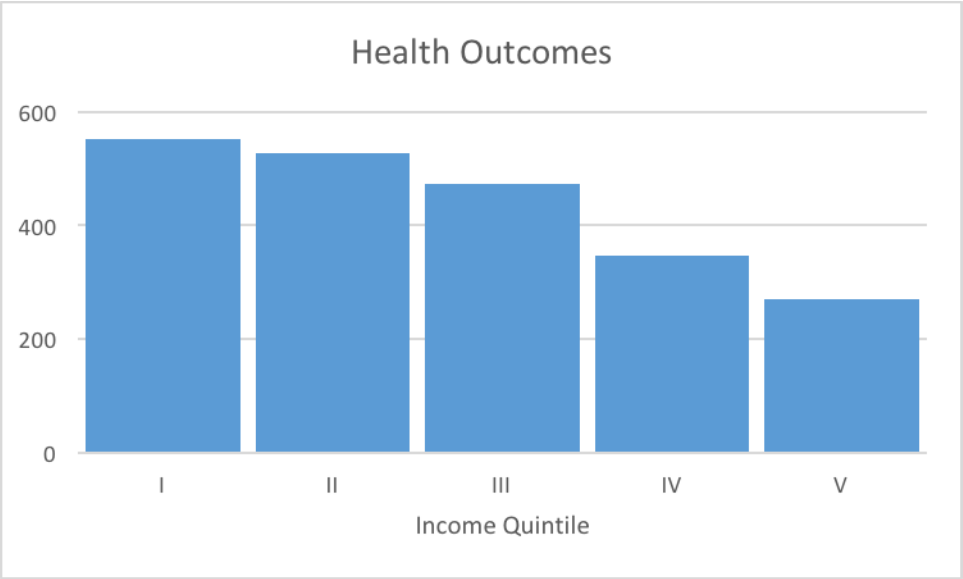

Figure S9: Anticipated benefits of a comprehensive helmet policy when substantially decreased by substandard helmets

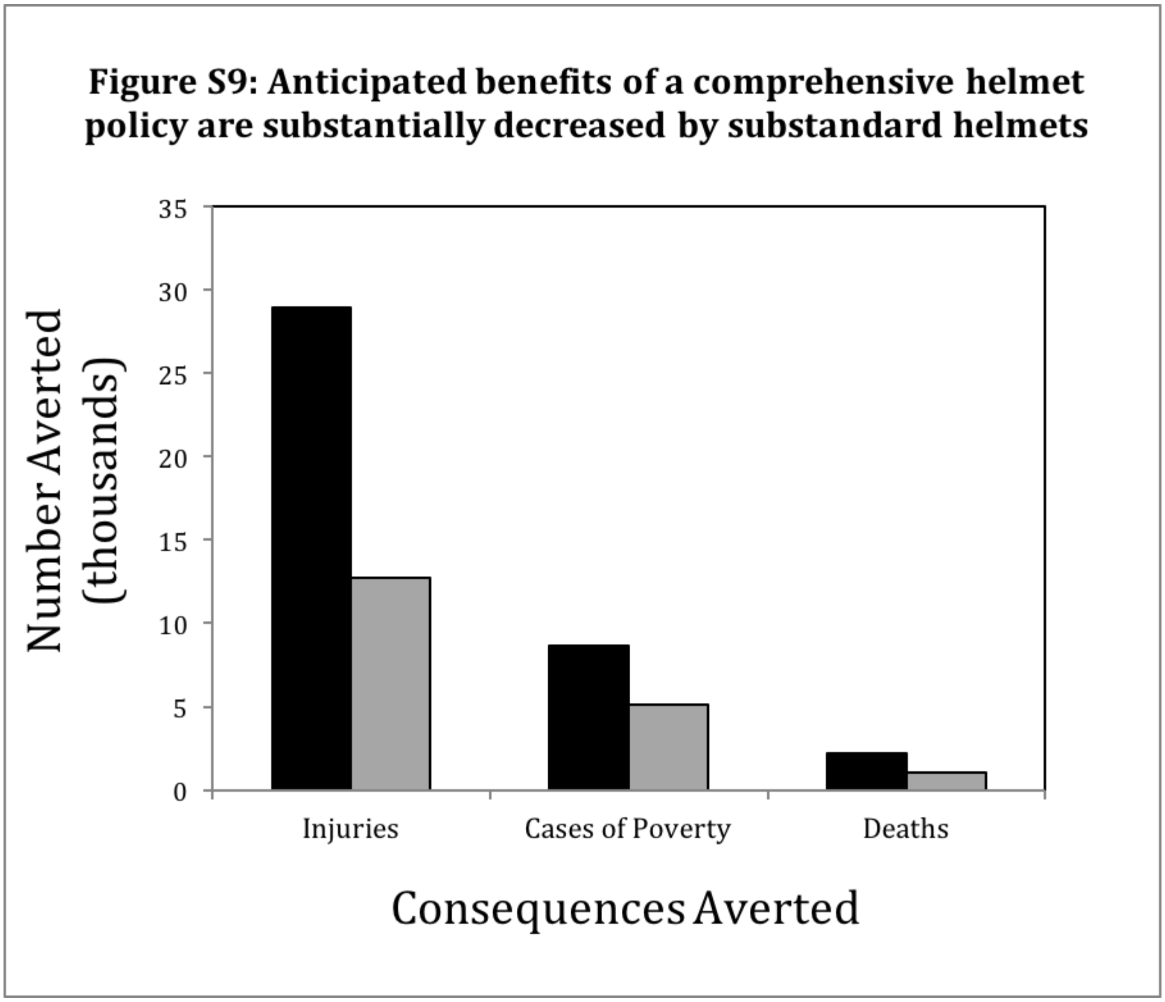

Legend: Simulation results from the main analysis shown in black. Simulation results from the sensitivity analysis assuming population-level helmet effectiveness reduced by the proliferation of substandard helmets shown in grey. Anticipated benefits from the comprehensive helmet policy are substantially decreased by substandard helmets.
